# Unraveling sub-seasonal precipitation variability in the Middle East via Indian Ocean sea surface temperature

**DOI:** 10.1038/s41598-024-53677-x

**Published:** 2024-02-05

**Authors:** Assaf Hochman, Noam Shachar, Hezi Gildor

**Affiliations:** Fredy and Nadine Hermann Institute of Earth Sciences, The Hebrew University of Jerusalem, Jerusalem, Israel

**Keywords:** Climate change, Climate sciences, Atmospheric science, Atmospheric dynamics

## Abstract

This study examines sub-seasonal precipitation anomalies, challenging to predict yet vital for society and the environment. Focusing on October, we investigate correlations between the Indian Ocean Dipole Mode Index (DMI), West Tropical Indian Ocean Index (WTIO), and Middle Eastern precipitation. We find robust correlations (~ 0.7), up to a two-month lag, demonstrating strong links between these climate indices and rainfall patterns, potentially suggesting sub-seasonal precipitation predictability. Over the past four decades, DMI and WTIO have shown a significant upward trend of ~ 0.4 °C, intensifying their impact on precipitation dynamics. This trend signifies evolving Indian Ocean climate patterns with potential regional consequences and is projected to continue in the twenty-first century. Significant correlations also emerge between DMI, WTIO, and maximum daily precipitation, highlighting their role in extreme rainfall events. Finally, our study attributes most of October’s precipitation variability to Indian Ocean sea surface temperature variations. These temperature anomalies influence the Indian Ocean’s Walker circulation, affecting water vapor flux to the Middle East and shaping regional precipitation. Our findings underscore the importance of these indices in understanding and predicting Middle East climate variability, revealing intricate ocean–atmosphere interactions.

## Introduction

Sub-seasonal precipitation predictability, a critical aspect of climate science, plays a pivotal role in understanding and forecasting weather patterns beyond a few days’ typical weather forecast horizon^1–3^. It encompasses the prediction of rainfall and its distribution over a period ranging from two weeks to a few months^4^. The importance of sub-seasonal predictability lies in its potential to address pressing societal and environmental challenges^5^. Accurate forecasts can significantly impact disaster preparedness, water resource management, and agriculture^6^. Timely knowledge of sub-seasonal precipitation patterns enables us to anticipate and mitigate the consequences of droughts, floods, and other extreme weather events, helping governments and communities make informed decisions^7^. Furthermore, it aids in optimizing resource allocation, ensuring food security, and fostering sustainable land use practices, particularly in vulnerable regions located on the border between temperate and arid climates, such as the Middle East.

Middle Eastern precipitation projections depict a region undergoing significant climatic transformations^8^. Projections based on climate models and observations indicate a general trend of increasing aridity in many parts of the Middle East, with reduced annual precipitation and prolonged drought conditions^8^. This shift is primarily attributed to rising global temperatures, altered atmospheric circulation patterns, and increased evaporation rates^9,10^. These changes pose substantial challenges to a region already characterized by water scarcity, potentially exacerbating water resource stress, agricultural challenges, and socioeconomic vulnerabilities^8^. However, it is important to note that the impact of climate change on precipitation can vary spatially within the Middle East, with some areas experiencing more pronounced declines in rainfall. In contrast, others may see relatively stable or even increased precipitation^11^. In this respect, the projected increase in SSTs may be an integral component of projected precipitation variability. Accurate and region-specific projections are essential for informed mitigation and adaptation strategies in this evolving climate challenge^12^.

Precipitation patterns in the Middle East exhibit remarkable variability across the four distinct seasons, driven by a complex interplay of synoptic conditions^13^. In winter, the region experiences an influx of moisture-laden air masses originating from the Mediterranean Sea, resulting in significant rainfall and occasional snowfall, especially in the northern and coastal areas^14^. Spring marks a transition period, with sporadic convective storms linked to the frontal boundaries moving in from the west. Summer ushers in a contrasting scenario characterized by the development of a subtropical high-pressure system dominating the region^15^. This pattern severely limits precipitation, causing arid conditions in most parts, while occasional monsoon-related showers influence the southern regions in June^16^. As autumn approaches, the subtropical high gradually weakens, allowing for sporadic rainfall, primarily affecting the southeastern parts of the Middle East in October^17^.

A complex interplay of geographical, meteorological, and climatic factors governs precipitation climatology in the Middle East during autumn^17^. This transitional period, from the dry and intense summer heat to more moderate temperatures, marks the onset of the region's rainy season^18^. The Mediterranean Sea serves as the principal moisture source, as humid air masses from the sea converge with the prevailing arid continental air^19^. This juxtaposition gives rise to diverse precipitation patterns, ranging from isolated convective thunderstorms to more extensive stratiform rainfall events^20^. Precipitation quantities exhibit notable spatial heterogeneity, with coastal zones experiencing more rainfall than inland areas. Autumn precipitation is paramount for agriculture, providing a critical water source for crops and ecosystems adapted to this seasonal variability^21^. A comprehension of autumn precipitation climatology in the Middle East is pivotal for informed water resource management, agricultural planning, and the advancement of regional climate science, given its direct ramifications on the socio-economic and environmental stability of the region^8^. An analysis recently found that the heat content in the upper layer of the eastern Mediterranean Sea in autumn significantly affects the following winter's precipitation amounts in various cities in Israel^22^. Therefore, monitoring ocean processes, mainly upper ocean Sea Surface Temperature (SST), may contribute to precipitation predictability in the region.

The Indian Ocean Dipole (IOD) and West Tropical Indian Ocean SSTs are integral components in the complex climate system of the Indian Ocean region^23^. The IOD is characterized by a gradient in SSTs between the western and eastern parts of the Indian Ocean and plays a key role in modulating regional climate patterns^24,25^ and sub-seasonal to seasonal scale forecasts^26–28^. When the western basin experiences warmer SSTs than the east basin, it results in a positive IOD phase. This temperature gradient influences the atmospheric circulation, leading to altered rainfall patterns across the Indian subcontinent and surrounding regions^29^. During a positive IOD event, there is an increased likelihood of above-average rainfall in the western Indian Ocean, including the Arabian Sea and eastern Africa^30–34^. In contrast, drier conditions and suppressed rainfall are often observed over the eastern Indian Ocean and Southeast Asia^35^. Conversely, the SST gradient reverses during a negative IOD phase, leading to contrasting precipitation anomalies. Climate models and observational data suggest that the IOD may undergo alterations in frequency and intensity. While projections vary, a consensus is that rising SST associated with global warming may lead to more frequent and stronger positive IOD events^36^. These changes have wide-ranging consequences, affecting regional rainfall patterns and extreme weather events in the Indian Ocean region. However, the role of the IOD in controlling Middle Eastern precipitation has yet to be studied.

The intricate interplay among the IOD, South Asian High, the monsoon system, and other large-scale circulation patterns has become a focal point of contemporary atmospheric science research^37,38^. Positive or negative phases of the IOD trigger discernible atmospheric changes with far-reaching implications for the region's climate dynamics^39^. Studies have demonstrated that during positive IOD events, there is a compelling association with a strengthened and northward-shifted South Asian High. This alteration in the atmospheric circulation patterns, influenced by both the IOD and South Asian High, sets the stage for intensified moisture transport from the Indian Ocean to the subcontinent^24^. Other studies underscore how these interconnected dynamics significantly impact the monsoon’s onset, duration, and intensity, thus influencing regional precipitation patterns^33^. Understanding these complex relationships is paramount for advancing sub-seasonal prediction capabilities^40^.

Integrated Water Vapor Flux (IWV) is fundamental in studying atmospheric moisture and its role in regulating precipitation^41^. IWV represents the total amount of water vapor flux within a column of the atmosphere. It is crucial in modifying precipitation patterns because it is a primary driver of moisture availability for rainfall events, mainly in extratropical regions such as the Middle East^42^. A high IWV indicates an abundant supply of atmospheric moisture, which can be a key factor in forming heavy rainfall and storms. Conversely, a low IWV can lead to drier conditions and reduced precipitation. The scientific community often uses IWV as a diagnostic tool to assess the potential for extreme precipitation events, particularly in regions prone to sub-seasonal to seasonal variability in rainfall, like the Middle East. Understanding and monitoring IWV is essential for improving weather forecasting and managing water resources, as it provides insights into the moisture dynamics that influence the timing, intensity, and spatial distribution of precipitation^43^.

This paper delves into the relationship between Indian Ocean SSTs and Middle East precipitation anomalies, mainly focusing on October. First, we study the links between the Dipole Mode Index (DMI), which is calculated as the difference between the West Tropical Indian Ocean (WTIO) and South Eastern Indian Ocean SSTs, and monthly precipitation in the Middle East (see “Methods”). For comparison, we further study the link between WTIO and precipitation anomalies. Then, we explain this link via circulation pattern differences and IWV variations. Finally, we provide nuanced projections over the twenty-first century of DMI, WTIO, and Middle Eastern precipitation during October based on the 6th phase of the Coupled Model Intercomparison Project (CMIP6) multi-model ensemble (Table S1).

## Results

### The impact of Indian Ocean Sea Surface temperature on monthly precipitation

**Figure 1:**
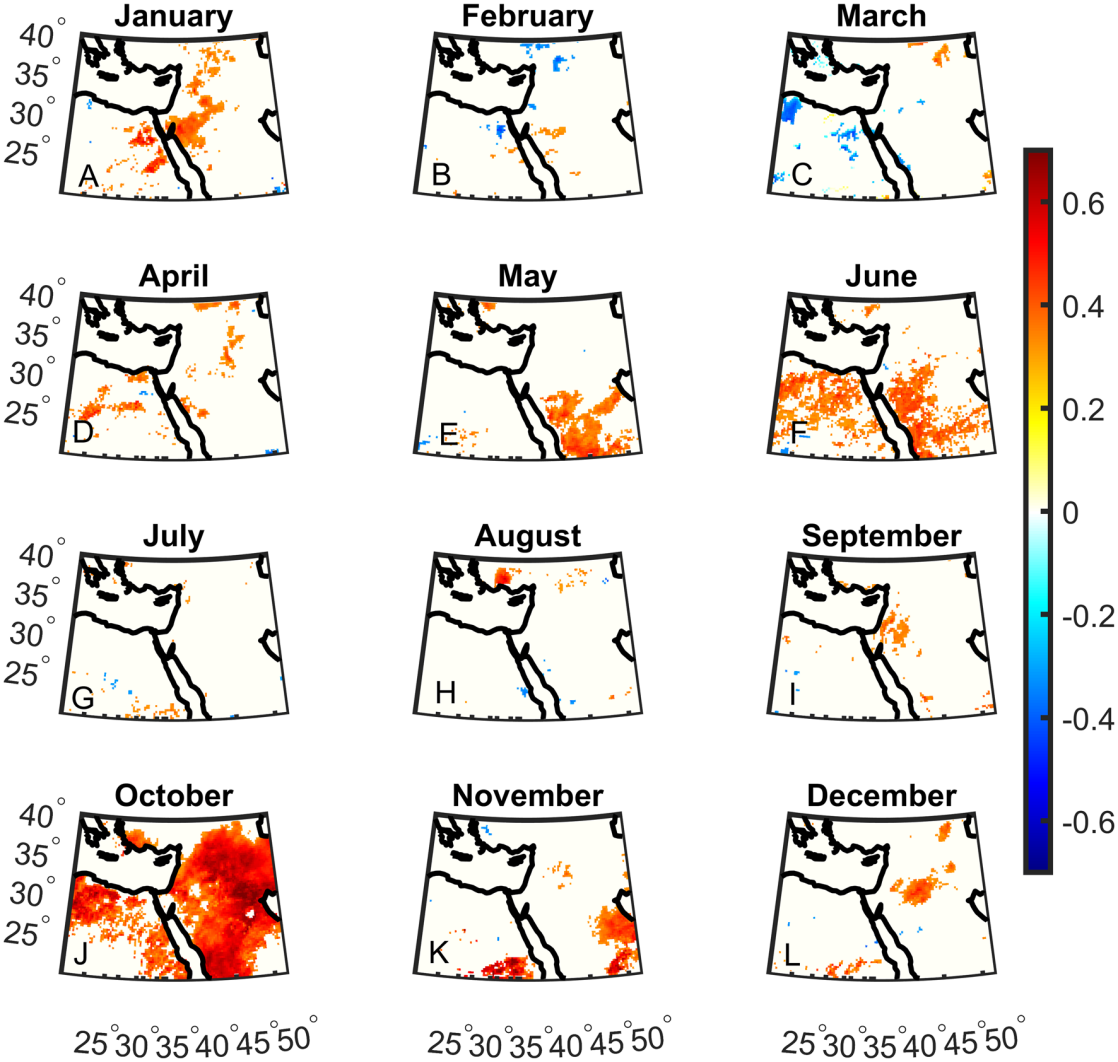
Monthly Dipole Mode Index (DMI) correlations with total precipitation over the Middle East. Significant correlations are shown in color using a student t-test at the 5% significant level. The correlation is most notable in October.

**Figure 2:**
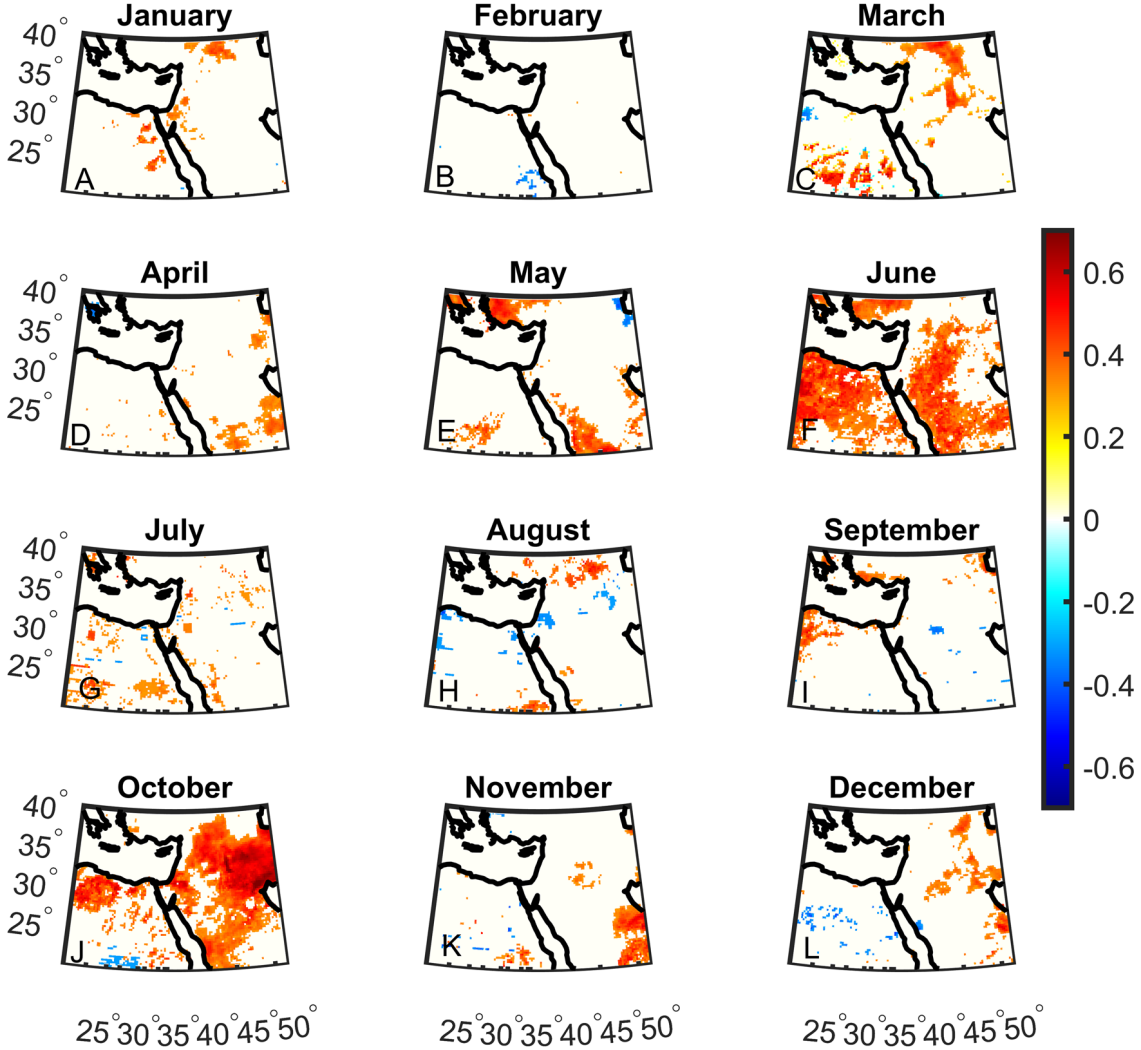
Same as Fig. 1 but for the West Tropical Indian Ocean Index (WTIO).

**Figure 3:**
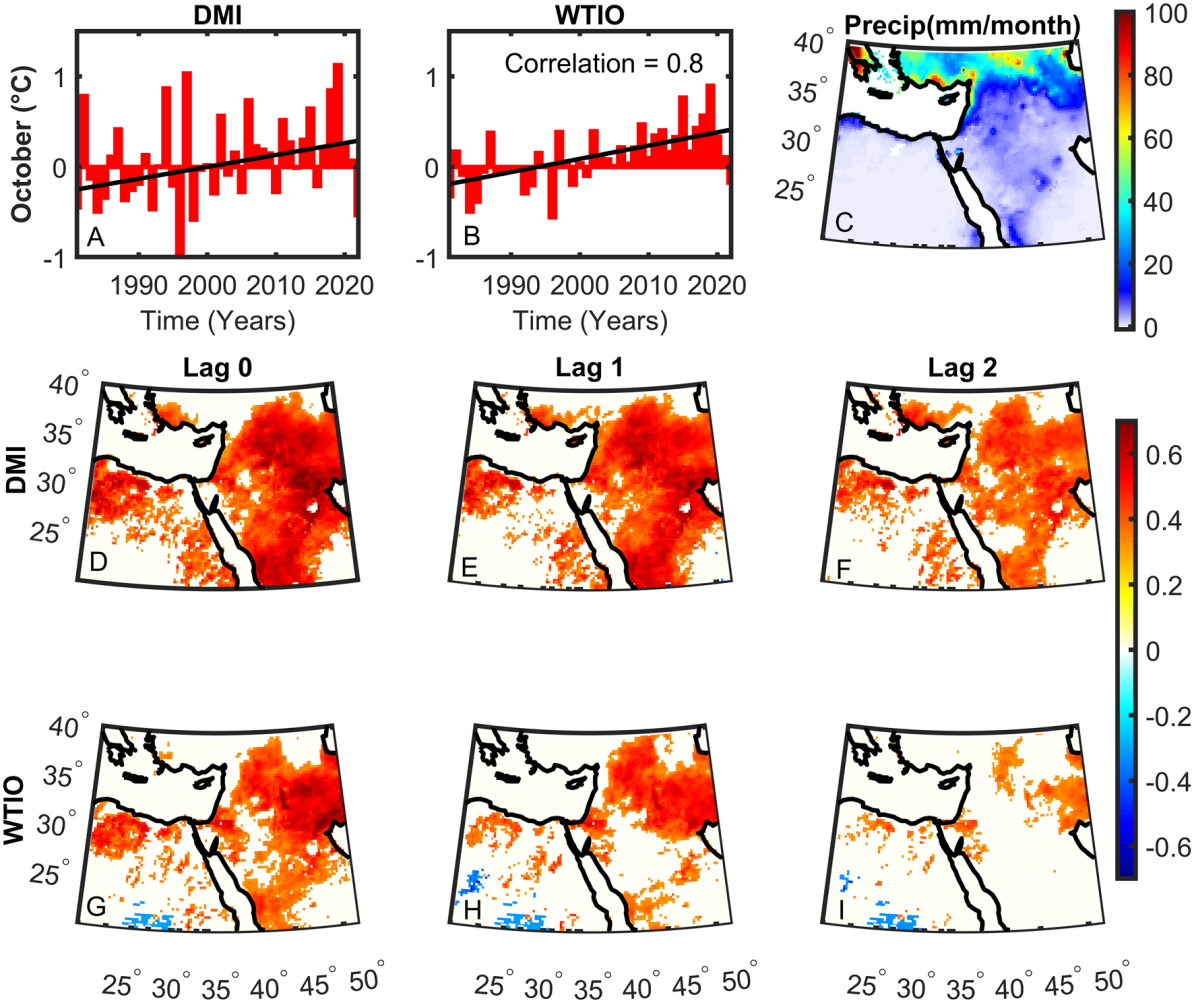
Dipole Mode (DMI) and West Tropical Indian Ocean (WTIO) indices for October (**A**, **B**). The correlation between DMI and WTIO is shown in panel (**B**). Precipitation climatology for October from 1981 to 2022 (C – mm/month). DMI (**D**–**F**) and WTIO (**G**–**I**) correlations with total October precipitation from 1981 to 2022 for lags 0 (**D**, **G**), 1 (**E**, **H**) and 2 (**F**, **I**). Significant correlations are shown in color using a student t-test at the 5% significant level.

First, we analyze the monthly correlations between DMI and precipitation anomalies over 1981–2022 (Fig. 1). Wide-spread significant correlations can be found mainly in October but also during January, May, and June (Fig. 1A, E, F and J). In the other months, some sporadic grid points show significant correlations. Indeed, during October, the subtropical high gradually weakens, allowing the Red Sea Trough to induce occasional intense rainfall events primarily affecting the southeastern parts of the Middle East. When considering the correlations between WTIO and precipitation anomalies, the correlations are less widespread and robust except for June, where strong and widespread correlations are exhibited (Fig. 2). Indeed, June is the transition period between precipitation still induced by cyclones arriving from the west in spring and the development of a subtropical high suppressing clouds and rainfall in summer. This allows for occasional monsoon-related showers influencing the southern regions during June. Based on these results, we focus our analysis on October, the transition month between the summer heat and the rainy season (see “Introduction”). October is characterized by precipitation climatology of ~ 0–100 mm, with most regions exhibiting ~ 0–40 mm (Fig. 3C).

**Figure 4:**
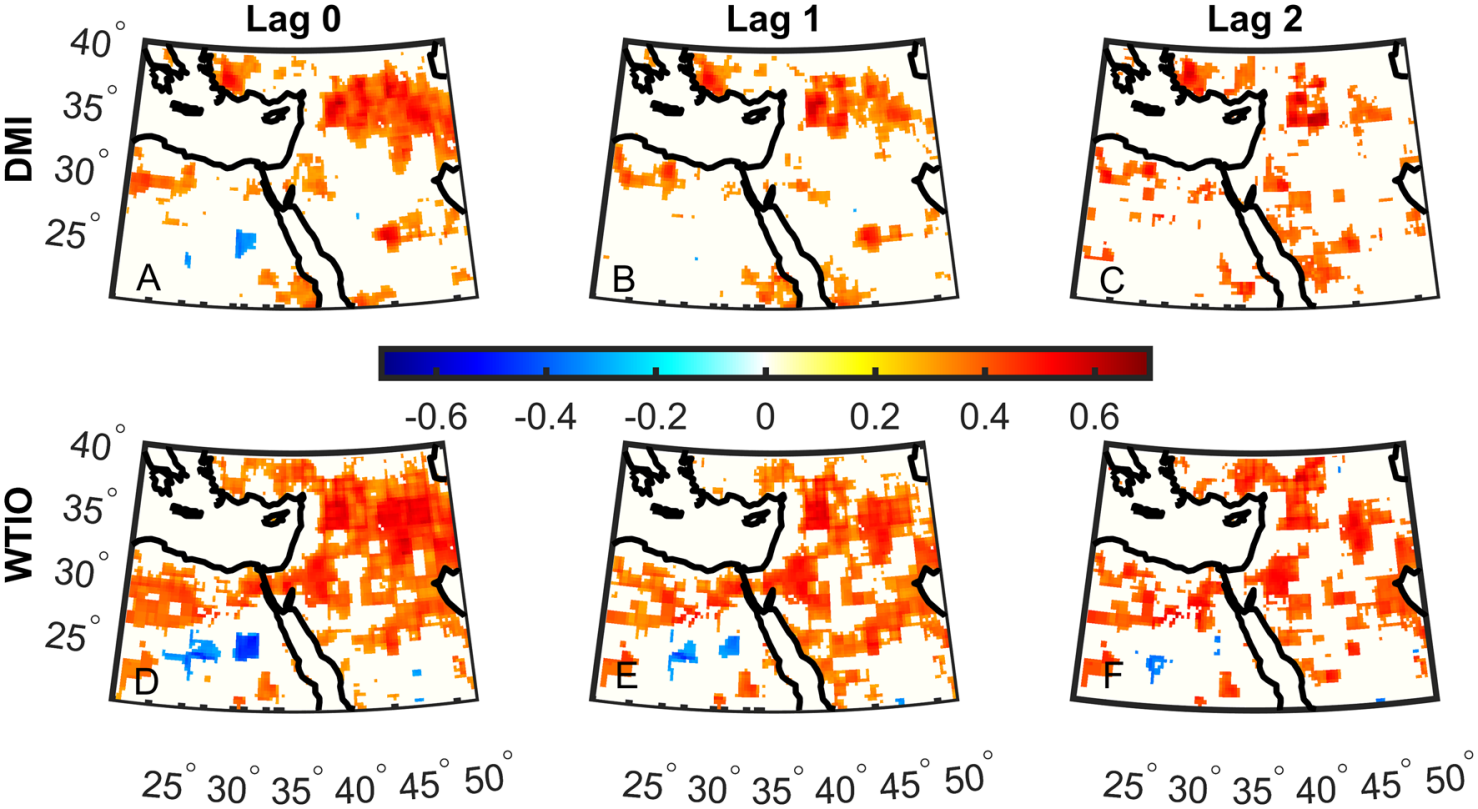
Same as Fig. 3D–I but for the maximum daily precipitation index (Rx1day).

Since 1981, a significant upward trend of ~ 0.4 °C is found in DMI and WTIO (Fig. 3A, B). The correlation between the two indices is 0.8, exemplifying the link between them though not a 1 to 1 relationship. This means that when the DMI is high, it is not necessarily that the WTIO is also high. This finding is further exerted when considering the lagged correlations between DMI, WTIO, and precipitation. We show that the correlations between DMI and precipitation are more substantial (Fig. 3D–F) compared to WTIO correlations (Fig. 3H–J). Indeed, the strength of the correlation reaches ~ 0.7 in large parts of the Middle East. The relatively strong correlations are kept in many regions for up to two months. Since we find strong correlations between the indices and total precipitation, we further test the correlations of DMI and WTIO with maximum daily rainfall in October (Rx1day; Fig. 4). The correlations are of the same order of magnitude as for total precipitation but now we show that for Rx1day the correlations are more substantial and more widespread for WTIO (Fig. 4D–F) than DMI (Fig. 4A–C). The above findings may suggest that the DMI controls total precipitation, whereas WTIO may have more control over extreme rainfall. Indeed, DMI is the difference in SST between the Western and Eastern parts of the Indian Ocean, thus strongly relates to the intensity of the Walker circulation. In contrast, WTIO may be high without increasing DMI. It should, however, be noted that correlations alone do not imply causality.

**Figure 5:**
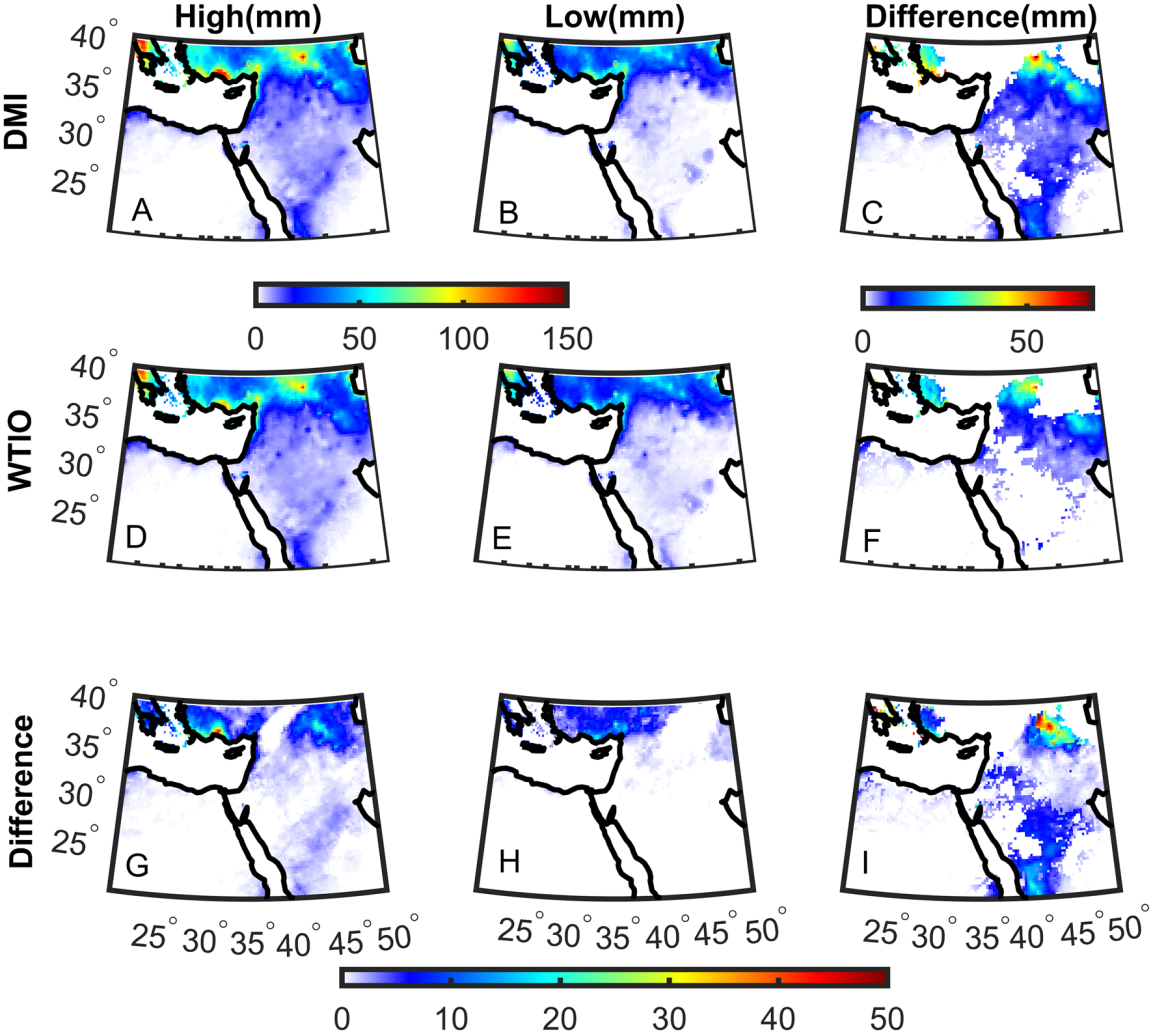
The total mean precipitation of the upper (High—**A**, **D**) and lower (Low—**B**, **E**) 20th percentile of the October Dipole Mode (DMI—**A**–**C**) and West Tropical Indian Ocean (WTIO—**D**–**F**) indices and the difference between ‘High’ and ‘Low’ (**C**, **F**). (**G**) The difference between (**A**) and (**D**). (**H**) The difference between (**B**) and (**E**). (**I**) The difference between (**C**) and (**F**). Significant differences using a bootstrap test at the 5% significance level are shown in color (**C**, **F**).

Finally, we quantify the influence that DMI and WTIO have on total precipitation (Fig. 5). We separate the distribution of DMI and WTIO into upper (high) and lower (low) 20th percentile subgroups to balance between having extreme values of the indices and retaining a large enough sample for robust statistical analysis. We find that total precipitation during October over large parts of the Middle East is significantly higher in the ‘high’ than the ‘low’ subgroup, reaching ~  + 60 mm in the mountainous regions of the ‘Fertile Crescent’ (Fig. 5C, F). The differences are more significant and more widespread for DMI than WTIO. Again, this suggests that DMI may have stronger control over total precipitation than WTIO. Examining the ‘high’ and ‘low’ months of DMI and WTIO. For the ‘high’ values of DMI and WTIO, we find that 50% of the years overlap, whereas for ‘low’ values, 75% overlap. Thus, although DMI and WTIO are strongly correlated (0.8), their ‘high’ and ‘low’ values do not necessarily overlap.

### Changes in circulation and water vapor flux influence monthly precipitation

**Figure 6:**
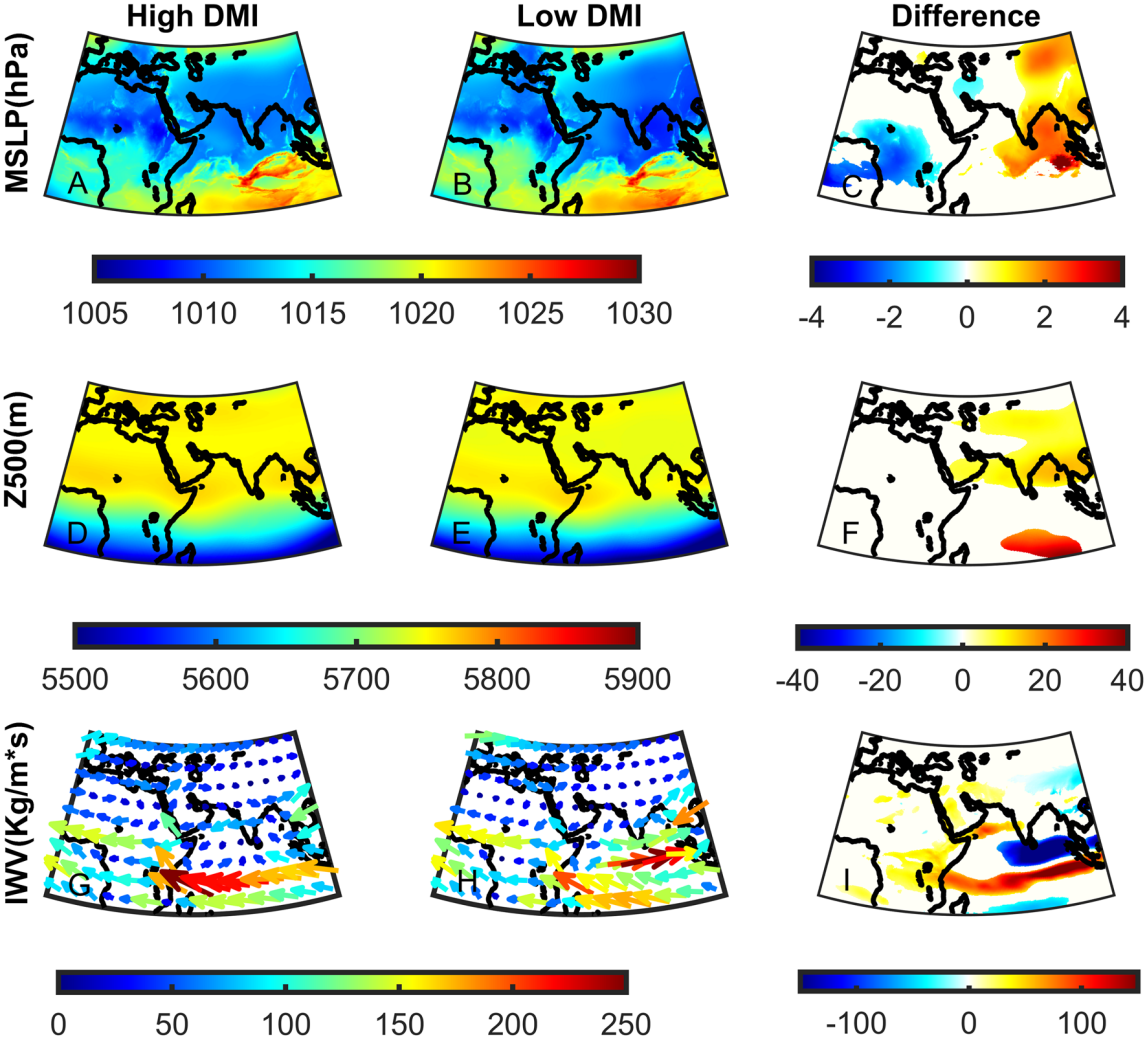
The upper (High—**A**, **D**, **G**) and lower (Low—**B**, **E**, **H**) 20th percentile of the October Dipole Mode Index (DMI) for Mean Sea Level Pressure (MSLP in hPa—**A–C**), 500hPa geopotential height (Z500 in m—**D**–**F**) and Integrated Water Vapor Flux (IWV in Kg/m*s—**G**–**I**). Significant differences are shown in color using a student t-test for MSLP and Z500 (**C**, **F**) and a bootstrap test for IWV (**I**) at the 5% significance level.

**Figure 7:**
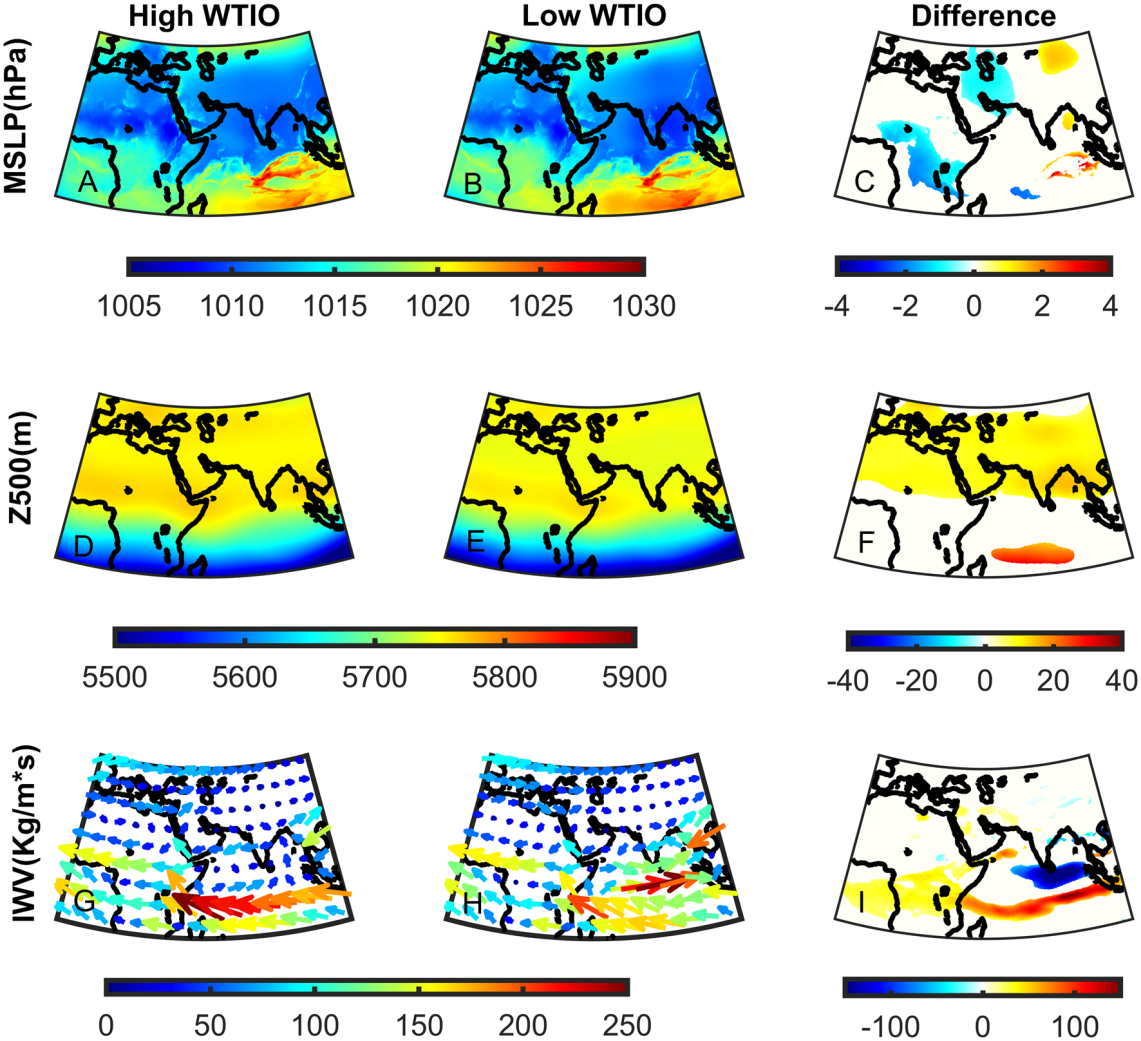
Same as Fig. 6 but for the West Tropical Indian Ocean Index (WTIO).

Next, we analyze how changes in DMI and WTIO influence the circulation patterns and water vapor flux to the Middle East (Figs. 6, 7). We analyze the differences in Mean Sea Level Pressure (MSLP), 500 hPa geopotential height (Z500), and IWV in the ‘high’ and ‘low’ DMI and WTIO subgroups. We find that during ‘high’ DMI, the Sudan Low is characterized by significantly lower pressure (~ − 4 hPa), and the region centered over the Bay of Bengal is characterized by substantially higher pressure (~ + 4 hPa; Fig. 6A–C). A deepening of the Sudan Low can also influence its northern branch, often termed the Red Sea Trough, the primary synoptic system bringing intense precipitation and thunderstorms to the region during October^44,45^. Moreover, such changes in surface-level circulation induce stronger pressure gradients and, thus, stronger south-easterly winds from the Indian Ocean towards the Middle East. The lower-level patterns (Fig. 6A–C) are further enforced by significantly higher Z500 over the Bay of Bengal and the Maldives, further intensifying the south easterly winds at upper levels (Fig. 6D–F). Complementary to the circulation changes, we find a significant difference in IWV patterns during ‘high’ DMI events (Fig. 6G–I). Indeed, there is a considerable increase in IWV from the Indian Ocean towards the eastern Mediterranean. This pattern suggests an increase in water vapor injected into the subtropical jet. Similar differences between ‘high’ and ‘low’ WTIO subgroups are shown, though with lesser magnitude than DMI subgroups (Fig. 7). This again suggests that DMI may have more substantial control over Middle East precipitation in October than the absolute WTIO SST values.

In summary, changes in DMI and WTIO strengthen the south easterly winds at both surface and upper levels, intensifying water vapor flux towards the Middle East. These differences may partly explain the significant differences in precipitation over the Middle East during ‘high’ Vs. ‘low’ DMI and WTIO events (Fig. 5).

### DMI, WTIO, and precipitation projections using CMIP6 multi-model ensemble

**Figure 8:**
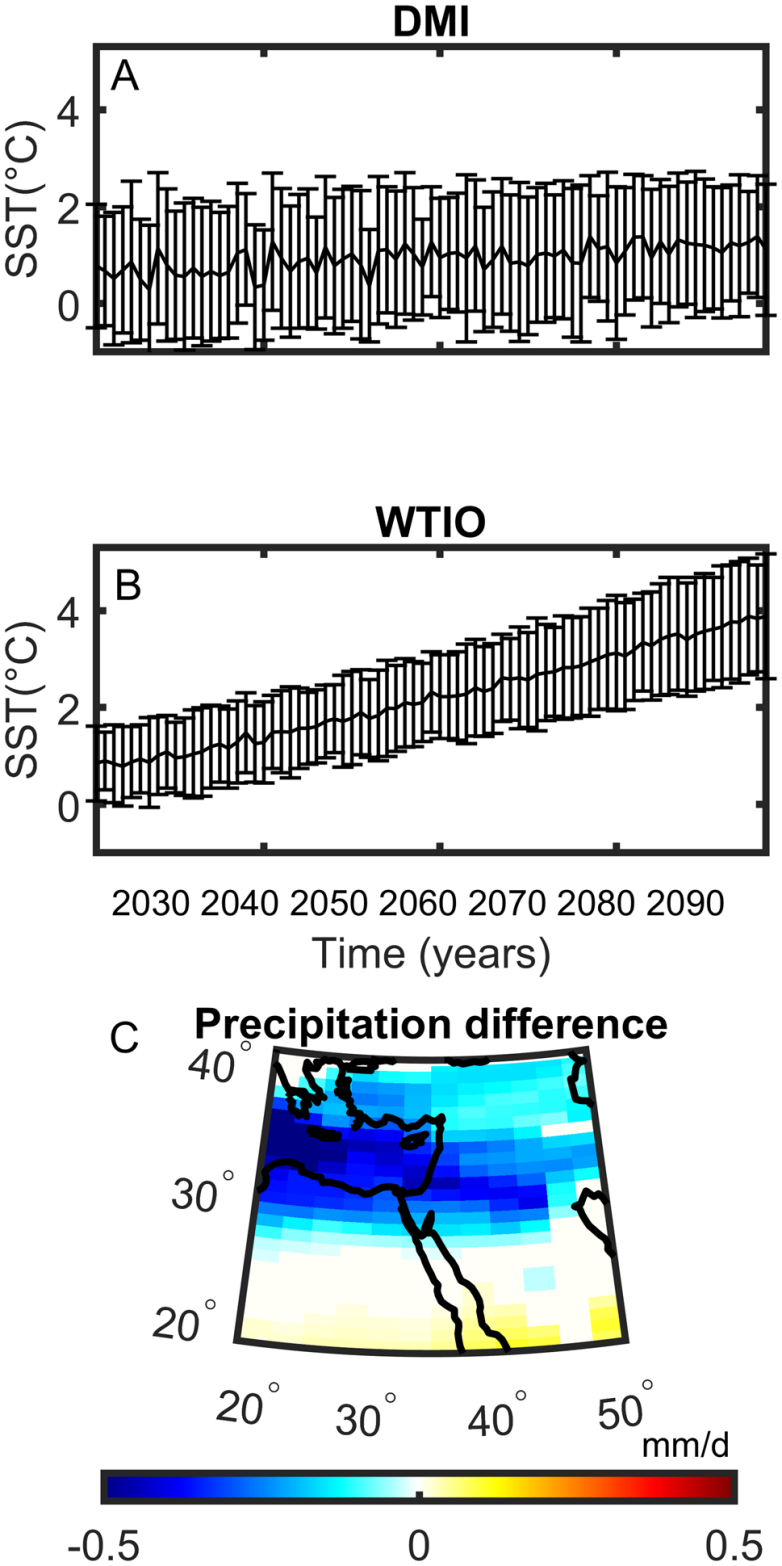
CMIP6 multi-model ensemble mean projections (SSP5-8.5) for October. (**A**) Dipole Mode Index (DMI) from 2023 to 2100 with one standard deviation error bars. (**B**) Same as (**A**) but for West Tropical Indian Ocean Index (WTIO). (**C**) The total precipitation difference (mm/day) between 2071–2100 and 1981–2010. Significant differences using a bootstrap test at the 5% significant level are shown in color. The list of models is in supplementary Table S1.

Expected projections of DMI and past variability have been studied before^46–48^. Fig. 8 presents the projected changes in DMI, WTIO, and Middle East precipitation during October based on the CMIP6 multi-model ensemble mean (SSP5-8.5). We find a significant projected increase in both DMI (~ + 0.6 °C) and WTIO (~ + 3 °C) by the end of the twenty-first century (Fig. 8A, B, respectively). Additionally, DMI and WTIO tend to stay positive throughout the century (Fig. 8A)^36^.

Finally, we display the October precipitation difference between the end of the twenty-first century (2071–2100) and the reference period (1981–2010; Fig. 8C). Still, the eastern Mediterranean region shows a significant decrease in total precipitation. However, significant increases in rainfall are shown in southerly locations of the domain that may relate, to some degree, to the rise in DMI and WTIO. No such increases are shown further north, which may be expected due to the projected rise in DMI and WTIO (Fig. 8C) and the strong control DMI and WTIO have on October precipitation in the Middle East (Figs. 1, 2, 3, 4 and 5). This finding may question the CMIP6 models' ability to simulate the interaction between Indian Ocean SST and Middle East precipitation in October, which we will explore in future studies. However, the decreasing precipitation trend in the northern part of the region could be influenced by factors originating from other basins, such as the Mediterranean storm track. At present, accurately simulating precipitation in climate models remains a considerable challenge.

## Summary and conclusions

The presented study explores the complex relationship between sub-seasonal precipitation anomalies in the Middle East, the Dipole Mode Index (DMI), and the West Tropical Indian Ocean Index (WTIO), mainly focusing on October. This research sheds light on the challenging yet vital task of predicting rainfall patterns, with significant implications for society and the environment. The findings demonstrate robust correlations between DMI, WTIO, and Middle Eastern precipitation, with correlation coefficients reaching approximately 0.7, even up to a two-month lag. These strong links indicate the influential role of these climate indices in shaping rainfall dynamics within the region. Indeed, transition months like October and June are linked with large-scale circulation changes, allowing for monsoon-related precipitation events whose moisture partly originates in the Indian Ocean. One of the prominent results of this study is the observed upward trend in both DMI and WTIO over the past four decades, with a temperature increase of approximately 0.4 °C. This trend is substantial as it suggests evolving Indian Ocean climate patterns, likely to have considerable regional consequences. The persistence of this trend into the twenty-first century (+ 0.6 °C for DMI and + 3 °C for WTIO) underscores the urgency of addressing the potential impacts of these evolving patterns on the Middle East's climate. Furthermore, this research reveals that DMI and WTIO correlate with overall precipitation patterns and extreme rainfall events, as evidenced by significant correlations with maximum daily precipitation. This stresses the importance of these indices in understanding the occurrence of intense and potentially disruptive weather events in the Middle East.

The study’s assertion that Indian Ocean SST variations largely account for October’s precipitation in the Middle East highlights the crucial role played by ocean–atmosphere interactions in shaping regional climate^22,49,50^. The connection between these temperature anomalies and the Indian Ocean’s Walker circulation directly influences water vapor flux to the Middle East, ultimately affecting the region's precipitation patterns.

As a caveat, we note that although we find significant correlations between Indian Ocean SSTs and Middle East precipitation anomalies, these correlations do not necessarily exert a causal relationship^51^.

This research contributes significantly to understanding the complex interactions between SST and Middle Eastern precipitation. It emphasizes the importance of considering these factors when predicting sub-seasonal climate anomalies^52^. It stresses the need to address the evolving climate patterns in the Indian Ocean, which have the potential to impact the Middle East’s climate in the coming decades^53^. This work is a valuable resource for policymakers, researchers, and climate scientists seeking to improve their understanding of Middle East climate variability and to develop more accurate prediction models for this vulnerable region.

### Data

We use the European Center for Medium-Range Weather Forecasting (ECMWF) ERA5 reanalysis from 1981 to 2022^54^. We extract averaged monthly Mean Sea Level Pressure (MSLP), 500 hPa geopotential height (Z500), and Northward and Eastward Integrated Water Vapor Flux (IWV) at 0.25° × 0.25° grid spacing in the domain (20 S–50 N; 10 W–110 E).

Reanalysis products cannot accurately depict convective precipitation, typical in arid areas such as the Middle East. It is, therefore, advisable to use alternative sources^55^. We employed precipitation data from the Climate Hazards Group InfraRed Precipitation with Station version 2 (CHIRPS)^56^, combining satellite infrared estimates with rain gauge information. We used CHIRPS daily precipitation estimates at 0.25° × 0.25° grid spacing from 1981 to 2022 in the domain (20 N–40 N; 20 E–50 E). Despite daily aggregation, CHIRPS' high-resolution infrared sensors on geostationary satellites^57^ effectively capture short-lived and localized convective systems typical during the autumn season in the Middle East^8,58^. CHIRPS is reliable and commonly used in climatological studies for regions with precipitation patterns analogous to the Middle East^59,60^.

The calculation of the Dipole Mode (DMI) and the West Tropical Indian Ocean (WTIO) indices is based on the Hadley Centre Global Sea Ice and Sea Surface Temperature version 1.1 (HadISST), which is a combination of monthly globally complete fields of SST and sea ice concentration for 1981–2022 at 1°× 1° grid spacing^61^. HadISST utilizes a reduced space optimal interpolation technique for SST data. It combines in-situ SST measurements with adjusted satellite-derived SST data.

We use the 6th phase of the multi-model ensemble of the Coupled Model Intercomparison Project (CMIP6) to project DMI, WTIO, and the Middle East October precipitation. CMIP6 represents the latest generation of climate models the scientific community uses to simulate and project Earth’s future climate^62^. These models are instrumental in understanding the complex interactions within the Earth’s climate system, including changes in SST and precipitation. CMIP6 models build upon their predecessors with improved spatial resolution, more comprehensive representations of Earth’s processes, and a broader range of scenarios for climate change. A list of the models used in this study is presented in supplementary Table S1.

## Methods

### Dipole Mode (DMI) and West Tropical Indian Ocean (WTIO) indices

The Dipole Mode (DMI) and the West Tropical Indian Ocean (WTIO) indices are climate indices that monitor SST anomalies in the Indian Ocean region^24^. DMI is typically calculated as the difference in SST anomalies concerning 1981–2010 mean climatology between two distinct areas within the Indian Ocean: the western pole (located around the Arabian Sea; 10 S–10 N; 50 E–70 E) and the eastern pole (positioned in the eastern equatorial Indian Ocean; 10 S–0; 90 E–108 E). It is expressed as DMI = SST (Western Pole)—SST (Eastern Pole). On the other hand, WTIO focuses only on the western pole SSTs and is calculated as the SST anomalies within that defined region.

### Calculation of integrated water vapor flux (IWV)

Integrated water vapor flux (IWV) is a crucial meteorological parameter used to assess the movement of water vapor within the atmosphere^41–43^. IWV is calculated by considering the horizontal and vertical components of the atmospheric water vapor flux within a specific region. The calculation involves the horizontal wind vector, the specific humidity profile, and the vertical extent of the atmosphere.

The IWV is mathematically expressed as the vertical integral of the product of the horizontal wind vector components (*u* and *v*) and the specific humidity (*q*):

$$IWV = \mathop \smallint \limits_{z1}^{z2} \left( {uq, vq} \right) dz$$

In this equation, u and v represent the zonal and meridional components of the horizontal wind vector. *q* is the specific humidity, which describes the amount of water vapor in the air. *z*1 and *z*2 denote the lower and upper boundaries of the vertical column, encompassing the atmosphere of interest. The integration is performed over the vertical extent of the atmosphere.

### Statistical inference

We used a set of statistical inference methodologies to unravel the relationship between DMI, WTIO, and Middle East precipitation.

1. We used spatial correlations between DMI, WTIO, and the Middle East total and maximum daily precipitation (Rx1day) at 0–2-month lags between the indices and precipitation. Significance testing at the grid point level was based on a student’s t-test at the 5% significance level (Figs. 1, 2, 3D–I, and 4).
2. We used the Theil-Sen slope estimator to calculate the observed and modeled trends in DMI and WTIO (Fig. 3A, B and Fig. 8A, B, respectively). The Theil-Sen estimator exhibits greater robustness when compared to the least-squares estimator due to its significantly reduced susceptibility to outliers^63,64^.
3. We quantified the precipitation, MSLP, Z500, and IWV differences between the upper and lower 20th percentiles of DMI and WTIO distributions. Significance testing at the grid point level was based on a student’s t-test at the 5% significance level for MSLP and Z500, which are considered to be distributed normally. A bootstrap test was used for IWV and precipitation that are not distributed normally (Figs. 5, 6 and 7).
4. The CMIP6 models were re-gridded to a common 1° × 1° before computing the differences in October precipitation between 2071–2100 and 1981–2010. Significance testing at the grid point level was based on a bootstrap test at the 5% significance level (Fig. 8C).

### Supplementary Information

Supplementary Table S1.

## Data Availability

The datasets used and analyzed during the current study are available from the corresponding author upon reasonable request.
