# Supplementary material for: Unraveling sub-seasonal precipitation variability in the Middle East via Indian Ocean sea surface temperature
Source: Sci Rep. 2024 Feb 5;14:2919. doi: 10.1038/s41598-024-53677-x (PMC10844372; doi:10.1038/s41598-024-53677-x)
Supplement: Supplementary file 1 — Supplementary Table S1. [file 41598_2024_53677_MOESM1_ESM.pdf]

Scientific Reports

Supplementary material for:

Unraveling Sub-Seasonal Precipitation Variability in the Middle East Via Indian Ocean Sea Surface Temperatures

Assaf Hochman[1*], Noam Shachar[1], Hezi Gildor[1]

*Corresponding author. Email: assaf.hochman@mail.huji.ac.il

1. Fredy and Nadine Hermann Institute of Earth Sciences, The Hebrew University of Jerusalem, Jerusalem, Israel.

Contents of this file:

Table S1

Table S1 List of Coupled Model Intercomparison Project phase-6 (CMIP6) models used in the analyses for both historical (1981-2010) and future (SSP5-8.5; 2071-2100) simulations. The table includes the following columns: model, grid spacing, and member.

**Model**	**Grid spacing (˚)**	**Member**
ACCESS-CM2	1.9 × 1.3	r1i1p1f1
CAMS-CSM1	1.1 × 1.1	r1i1p1f1
CanESM5	2.8 × 2.8	r1i1p2f1
CESM2	1.3 × 0.9	r4i1p1f1
CIESM	1.3 × 0.9	r1i1p1f1
CMCC-ESM2	0.9 × 1.25	r1i1p1f1
CNRM-CM6-1-HR	0.5 × 0.5	r1i1p1f2
EC-Earth3-CC	0.7 × 0.7	r1i1p1f1
FGOALS-f3-L	1.3 × 1	r1i1p1f1
FIO-ESM	1.3 × 0.9	r1i1p1f1
HadGEM3-GC31-LL	1.25 × 1.875	r1i1p1f3
IITM-ESM	1.89 × 1.89	r1i1p1f1
INM-CM5	2 × 1.5	r1i1p1f1
IPSL-CM6A-LR	2.5 × 1.3	r1i1p1f1
KIOST-ESM	1.89 × 1.89	r1i1p1f1
MCM-UA	2.25 × 2.25	r1i1p1f2
MIROC6	1.4 × 1.4	r1i1p1f1
MPI-ESM1-2-LR	1.9 × 1.9	r1i1p1f1
NESM3	1.9 × 1.9	r1i1p1f1
NorESM2-MM	2.5 × 1.9	r1i1p1f1
UKESM1-0-LL	1.9 × 1.3	r1i1p1f2
